# Clinical profile and electrophysiological characteristics of atypical atrioventricular nodal reentrant tachycardia: A decade's experience

**DOI:** 10.1016/j.ipej.2023.10.004

**Published:** 2023-10-14

**Authors:** Ashesh Halder, Soorampally Vijay, Yogesh Kolamkar, Yagnik Mukund Kumble, Yash Lokhandwala

**Affiliations:** a Department of Cardiology, Holy Family Hospital, Mumbai, India; b Department of Cardiology Medica Superspecialty Hospital, Kolkata, India

**Keywords:** Supraventricular tachycardia, Electrophysiology study, Arrhythmia

## Abstract

**Objective:**

To assess the clinical features and inducibility characteristics of atypical atrioventricular nodal reentrant tachycardia (AVNRT) and compare it with typical AVNRT.

**Background:**

AVNRT is the commonest form of paroxysmal supraventricular tachycardia. The mechanism of AVNRT is very varied. Several classification systems evolved with better understanding but a simplified approach of classification into typical and atypical AVNRT is justifiable and clinically more relevant. In our study, we have assessed the epidemiological profile of atypical AVNRT in a single institute over 10 years and analysed pertinent electrophysiological characteristics.

**Method:**

In this retrospective observational single center study we analysed data of all AVNRT cases from January 2011 to June 2021. In our study we classified atypical AVNRT and typical AVNRT based on the HA interval; HA≤70 ms in the His bundle region during tachycardia was considered as typical AVNRT. Other parameters were also analysed during tachycardia, such as: induction by atrial or ventricular pacing, AH/HA ratio, tachycardia cycle length and site of the earliest atrial activation. The demographic profile of the patients were also compared between 2 groups.

**Results:**

Atypical AVNRT was found in 75/1431 patients (5.2%) of all cases of AVNRT. The age of patients with atypical AVNRT was 52.4 ± 15.2 years (range 9–82 years) while that for typical AVNRT it was 48.2 ± 15.7 years (2–89 years), p = 0.023. There was no gender difference. Atypical AVNRT was induced by only ventricular extrastimuli (VES) in 17/75 (22.6%) while in typical AVNRT this was seen in only 12/1356 patients (0.9%, p < 0.001). Induction of atypical AVNRT was seen by both atrial extrastimuli (AES) and VES in 17/75 patients (22.6%) while in typical AVNRT this was seen in 64/1356 patients (4.8%, p < 0.001). Atypical AVNRT was induced by only AES in 40/75 patients (53.3%) while in typical AVNRT this was seen in 1280/1356 patients (94.3%, p < 0.001). An AH >200 ms during tachycardia was seen in all patients with typical AVNRT and in only 31/75 patients (41.3%) of atypical AVNRT (p < 0.00001). An interesting finding in atypical AVNRT was the earliest atrial activation at the His bundle region in 10/75 (13.3%) patients.

**Conclusion:**

Atypical AVNRT prevalence depends on the way it is classified; this was 5.2% of all AVNRT cases in our study. Typical AVNRT was seen more frequently in comparatively younger age group and was more often induced by AES. Atypical AVNRT was much more commonly induced by only VES compared to typical AVNRT. It was not so unusual in atypical AVNRT to find the earliest atrial activation in the His bundle region.

## 1 Introduction

The prevalence of supraventricular tachycardia in the general population is 2.25/1000 persons and it's yearly incidence is 35 per 100000 persons [7]. AVNRT is the most common mechanism responsible for symptomatic supraventricular tachycardia. Women are more predisposed and after the second decade AVNRT is more frequently seen than atrioventricular reentrant tachycardia or atrial tachycardia among patients undergoing radiofrequency ablation [8].

It has been more than 100 years since the conceptualization of reentry and more than half a century since the postulation of duality of the atrioventricular node, but the precise mechanism of different types of AVNRT is yet a work in evolution [4,5]. The classification of typical (slow-fast) and atypical AVNRT (fast-slow, slow-slow, slow-intermediate, etc.) was initially prevalent. But these subdivisions of atypical AVNRT were rather nebulous and overlapping. It was also proposed that in typical AVNRT the earliest ‘A’ was at the His bundle region while in atypical AVNRT the earliest ‘A’ was near the coronary sinus ostium (CS–Os) [1,6]. Later on it was found that i) a significant proportion of slow-fast AVNRT had the earliest atrial activation (‘A’) at the CS-Os) or the left atrium instead of the classical antero-superior location and ii) atypical AVNRT could have the earliest ‘A’ in the low right atrial septum, His bundle area or even left lateral area instead of the traditional CS-Os region [6,[8], [9], [10]]. From a more practical standpoint, the classification of AVNRT into typical and atypical based on the HA interval [6] is widely adopted and we have followed this classification system in our study.

## 2 Method

This is a retrospective observational single center study analysing all patients of AVNRT who underwent radiofrequency ablation from January 2011 to June 2021. Anti-arrhythmics were stopped four half-lives before the procedure. The procedure was done under local anaesthesia or rarely conscious sedation (small children). We used EP-TRACER (Cardio Tek, Maastricht, Netherlands) for recording. A decapolar catheter was placed within the CS, a quadripolar catheter was placed at the His bundle area and a mapping catheter was placed in the right atrium/right ventricle. During induction protocol AES was given first followed by VES. AES and VES were given during induction in all patients irrespective of tachycardia induction by either AES or VES. AH and HA intervals were measured during tachycardia, typical and atypical AVNRT was diagnosed after doing several maneuvers (response to atrial and ventricular overdrive pacing, His refractory VES, resetting response to PAC). A HA>70 ms in the His bundle region during tachycardia was considered as atypical AVNRT. Diagnosis of typical AVNRT was accepted when there was HA≤70 ms at His-bundle region. Ethical approval was received from our institution's ethics committee.

## 3 Data analysis

All patients’ data were analysed and represented as mean ± SD. Groups were compared using student t-test and Chi-Square test.

## 4 Results

During the stipulated period of 10 years, there were 1431 patients of AVNRT, of which 75 (5.2%) had atypical AVNRT. Atypical and typical AVNRT were coexistent in 18 patients. The age of patients with atypical AVNRT was 52.4 ± 15.2 years (9–82 years) compared to typical AVNRT where the age was 48.2 ± 15.7 years (2–89 years, p = 0.023). The female to male ratio was 1:1 in atypical AVNRT (female

38, male 37) while in typical AVNRT this was 1.4:1 (female 787, male 569), but this was not significantly different.

**Tachycardia induction:** Isoprenaline was required for induction in 13% patients; no drugs were required for induction in the others. Atypical AVNRT was induced by only VES in 17/75 (22.6%) patients compared to typical AVNRT which was induced by only VES in 12/1356 patients (0.9%, p < 0.001). Induction of atypical AVNRT was seen by both AES and VES in 17/75 patients (22.6%) compared to typical AVNRT where this was seen in 64/1356 patients (4.8%, p < 0.001). Atypical AVNRT was induced by only AES in 40/75 patients (53.3%) while in typical AVNRT this was seen in 1280/1356 patients (94.3%, p < 0.001) [Table 1].

**Table 1:** Age, gender and tachycardia characteristics. VES, ventricular extrastimuli; AES, atrial extrastimuli.

	Atypical AVNRT	Typical AVNRT	p value
**Patients**	75	1356	
**Age (years)**	52.4 ± 15.2	48.2 ± 15.7	0.023
**Gender**			0.2085
M	37	569	
F	38	787	
**Induction**			<0.001
Only VES	17	12	
VES and AES	17	64	
Only AES	40	1280	
Tachycardia cycle length (ms)	354 ± 64	320 ± 49	0.000027
AH>200	31 (41.3%)	1356 (100%)	<0.00001

Atypical AVNRT	patients
**AH≤200**	39 (52%)
AH/HA>1	11 (14.7%)
AH/HA<1	28 (37.3%)
**Earliest atrial activation**	
1 CS-Os	47(62.7%)
2 CS Os and His	14(18.7%)
3 His	10 (13.3%)
4 Mid CS	1(1.3%)

**N.B**: Atypical AVNRT was present in 75 patients but we had few missing data [:a) 1 for mode of tachycardia induction; b) 5 in respect with tachycardia cycle length and AH interval; c) 3 for site of early atrial activation].

**Fig. 1:**
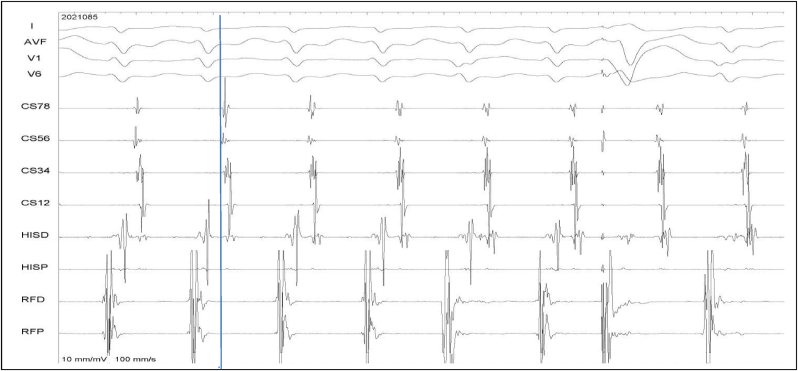
Atypical AVNRT, commonest variety. AH 178 ms and HA 208 ms. Here the retrograde atrial activation was earliest at CS-Os. A His-refractory ventricular extrastimulus did not change the next atrial timing or sequence.

**Fig. 2:**
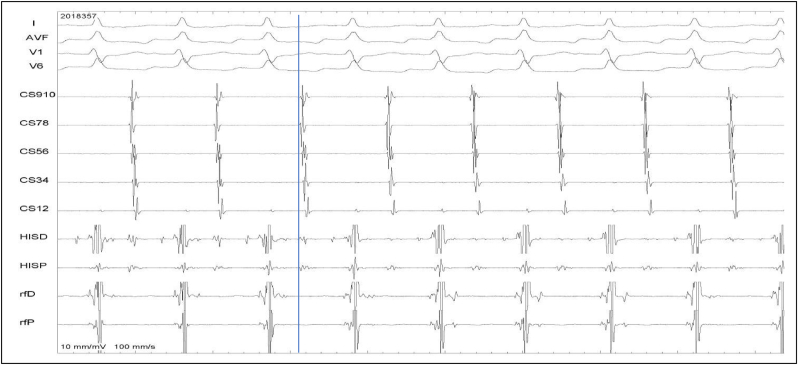
Atypical AVNRT, less common variety. HA 211 ms and AH 111 ms. Retrograde atrial activation was simultaneously early at His and CS-Os.

**Fig. 3:**
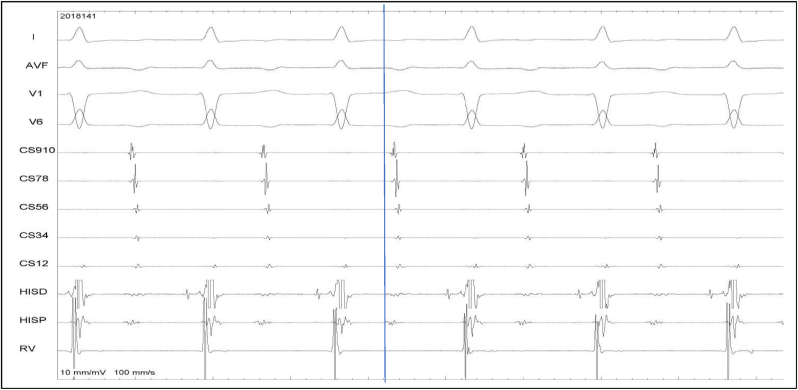
Atypical AVNRT, uncommon variety. AH is 198 ms and HA is 214 ms. The atrial activation was earliest at the His region.

**Fig. 4:**
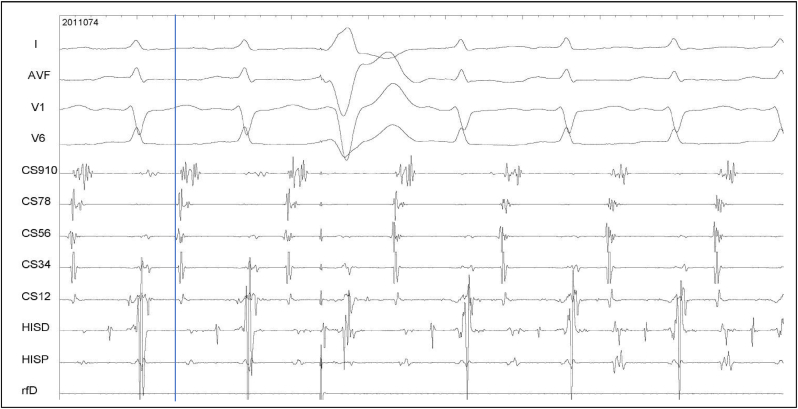
Atypical AVNRT, rare variety. AH is 102 ms and HA is 218 ms. The atrial activation was earliest in the mid CS region (like “bracketing”) mimicking an accessory pathway. A His-refractory ventricular extrastimulus from the right ventricular apex did not affect the next atrial activation.

**Tachycardia characteristics:** The tachycardia cycle length in atypical AVNRT was 354 ± 64 ms compared to 320 ± 49 ms in typical AVNRT (P = 0.000027). The earliest ‘A’ was seen at the CS-Os in atypical AVNRT in 47/75 (62.7%) patients, simultaneously early at CS-Os and His bundle area in 14/75 (18.7%) patients, at the His bundle area in 10/75 (13.3%) patients and inside the CS in 1 patient. An AH >200 ms was seen in 31/75 (41.3%) of atypical AVNRT and in all patients with typical AVNRT (p < 0.00001). In 39/75 (52%) cases of atypical AVNRT the AH was ≤200 ms, of which AH/HA>1 was seen in 11/75 (14.7%) and AH/HA<1 was seen in 28/75 (37.3%) patients.Those with longer HA interval (mean HA 176 ms) had earliest activation at or closer to CS-Os. Mean HA interval was 108 ms in those who had earliest “A” at His. Mean HA was 153 ms in subgroup of the atypical AVNRT induced only by AES, whereas mean HA was 207 ms when typical AVNRT was induced by only VES [Fig. 1, Fig. 2, Fig. 3, Fig. 4, Table 1].

**Radiofrequency ablation:**All patients of atypical AVNRT underwent successful ablation either in the slow pathway region or by targeting the earliest retrograde atrial activation. It was not unusual to find loss of VA conduction while getting junctional acceleration during ablation in atypical AVNRT. So, in many occasions short burst of RF energy was given. Sometimes, RF energy was given with atrial pacing as soon as there was loss of VA conduction with junctional acceleration to ascertain normal antegrade AV nodal conduction.

## 5 Discussion

The prevalence of atypical AVNRT was 5.2% in our study which is quite similar with the recent study of Katritsis et al. [1] wherein this was 6.4%, but it is different from other older studies (slow/fast 81.4%, slow/slow 13.7%, fast/slow 4.9%), mostly because of different classification approaches [2]. While some authors classified atypical AVNRT based on the VA or HA time [1,6] (again, there are ambiguities regarding whether the ‘A’ was considered only at the His bundle region or even down to the CS-Os), others considered the AVNRT to be atypical if the earliest ‘A’ was at a site other than the His bundle region, especially at the CS-Os [9,10]. The approach to AVNRT classification has been described broadly as that of the ‘splitters’ and the ‘lumpers’. The splitter approach was to subclassify atypical AVNRT as fast-slow, slow-slow, slow-intermediate, etc. An antegrade slow pathway was considered as AH>200 ms, a retrograde slow pathway was considered if the HA≥70 ms^8^. We preferred the lumper approach, using a cut-off of HA>70 ms at the His bundle area to diagnose atypical AVNRT.

Though the age of presentation in atypical AVNRT was a little higher compared to typical AVNRT, there was considerable overlap. We didn't have any gender preponderance in atypical AVNRT, several studies have shown a definite female predominance in both typical and atypical AVNRT [3,11]. Atypical AVNRT was induced with VES more often than typical AVNRT, which was quite expected. An intriguing part of our study was observation of earlier retrograde atrial activation site at locations other than the traditional CS-Os in almost 34% patients of atypical AVNRT. Various studies have also shown that the early atrial activation site in atypical AVNRT could be at the CS-Os, right midseptal area, His bundle region and inside the CS (even at the left lateral region) [6,9,10].

All the patients of atypical AVNRT in our study underwent successful ablation either in the slow pathway region or by targeting the earliest retrograde atrial activation.

Katritsis et al. [11] have shown a good outcome of atypical AVNRT ablation by targeting the anatomical slow pathway region.

## 6 Conclusion

The prevalence of atypical AVNRT is related to the classification system. In our study, the age of presentation was higher and tachycardia cycle length was longer in atypical AVNRT compared to typical AVNRT.Atypical AVNRT was more commonly induced by ventricular extrastimuli.It was not so unusual in atypical AVNRT to find the earliest atrial activation in the His bundle region.

## Limitations

We lost some data of few patients of atypical AVNRT as mentioned in Table 1

## Grants, contracts, and other forms of financial support

None

## Funding

Nil

## Declaration of competing interest

The authors declare that they have no known competing financial interests or personal relationships that could have appeared to influence the work reported in this paper.
